# A high-density genetic map for anchoring genome sequences and identifying QTLs associated with dwarf vine in pumpkin (*Cucurbita maxima* Duch.)

**DOI:** 10.1186/s12864-015-2312-8

**Published:** 2015-12-24

**Authors:** Guoyu Zhang, Yi Ren, Honghe Sun, Shaogui Guo, Fan Zhang, Jie Zhang, Haiying Zhang, Zhangcai Jia, Zhangjun Fei, Yong Xu, Haizhen Li

**Affiliations:** Beijing Vegetable Research Center, BAAFS, Key Laboratory of Biology and Genetic Improvement of Horticultural Crops (North China), Ministry of Agriculture, Beijing Key Laboratory of Vegetable Germplasm Improvement, Beijing, 100097 P.R. China; Boyce Thompson Institute for Plant Research, Cornell University, Ithaca, NY 14853 USA; USDA-ARS, Robert W. Holley Center for Agriculture and Health, Ithaca, NY 14853 USA

**Keywords:** High-density genetic map, Pumpkin (*Cucurbita maxima* Duch.), Scaffold anchoring, QTL mapping, Dwarf vine

## Abstract

**Background:**

Pumpkin (*Cucurbita maxima* Duch.) is an economically important crop belonging to the Cucurbitaceae family. However, very few genomic and genetic resources are available for this species. As part of our ongoing efforts to sequence the pumpkin genome, high-density genetic map is essential for anchoring and orienting the assembled scaffolds. In addition, a saturated genetic map can facilitate quantitative trait locus (QTL) mapping.

**Results:**

A set of 186 F_2_ plants derived from the cross of pumpkin inbred lines Rimu and SQ026 were genotyped using the genotyping-by-sequencing approach. Using the SNPs we identified, a high-density genetic map containing 458 bin-markers was constructed, spanning a total genetic distance of 2,566.8 cM across the 20 linkage groups of *C. maxima* with a mean marker density of 5.60 cM. Using this map we were able to anchor 58 assembled scaffolds that covered about 194.5 Mb (71.7 %) of the 271.4 Mb assembled pumpkin genome, of which 44 (183.0 Mb; 67.4 %) were oriented. Furthermore, the high-density genetic map was used to identify genomic regions highly associated with an important agronomic trait, dwarf vine. Three QTLs on linkage groups (LGs) 1, 3 and 4, respectively, were recovered. One QTL, *qCmB2*, which was located in an interval of 0.42 Mb on LG 3, explained 21.4 % phenotypic variations. Within *qCmB2*, one gene, *Cma_004516*, encoding the gibberellin (GA) 20-oxidase in the GA biosynthesis pathway, had a 1249-bp deletion in its promoter in bush type lines, and its expression level was significantly increased during the vine growth and higher in vine type lines than bush type lines, supporting *Cma_004516* as a possible candidate gene controlling vine growth in pumpkin.

**Conclusions:**

A high-density pumpkin genetic map was constructed, which was used to successfully anchor and orient the assembled genome scaffolds, and to identify QTLs highly associated with pumpkin vine length. The map provided a valuable resource for gene cloning and marker assisted breeding in pumpkin and other related species. The identified vine length QTLs would help to dissect the underlying molecular basis regulating pumpkin vine growth.

**Electronic supplementary material:**

The online version of this article (doi:10.1186/s12864-015-2312-8) contains supplementary material, which is available to authorized users.

## Background

*Cucurbita maxima* including pumpkin, hubbard, turban and buttercup squash, is native to South America and belongs to the genus *Cucurbita* L. with *n* = 20 chromosomes [1]. Pumpkin is one of the most economically important crops within this species [2], and commonly known as winter squash with its mature fruits consumed as vegetables in most of the world, especially in Asia (primarily China and India) and Africa. Even in developing countries, pumpkin is a staple food and a rich source of fat, iron, calcium and vitamins [3]. In addition, pumpkin seeds are also used as food, because they are excellent sources of proteins (32-44 %), oil (34-50 %, over 70 % of unsaturated fatty acids comprised mainly by linoleic and oleic acids) and vitamin E [4, 5]. The interspecific hybrids of *C. maxima* × *C. moschata* are important rootstocks of cucumber and watermelon to increase crop disease resistance, stress-tolerance and yield, and improve fruit quality [6–8].

Despite its economic importance, there are very limited genomic and genetic resources available for *C. maxima*; unlike other cucurbits, such as cucumber, watermelon and melon, even *C. pepo* and *C. moschata*, for which dense genetic maps [9–14], microarrays [15, 16], reverse genetic platforms [17–19], transcriptomes [20–25] and even whole genome sequences [26–28] have been developed and generated. These resources have provided powerful tools for genetic and genomic analysis in exploring gene functions and genetic diversities, and facilitating classical linkage mapping and association mapping, and breeding new varieties [29–36].

Saturated genetic linkage maps are critical for assembled genome scaffold anchoring and orienting, quantitative trait locus (QTL) mapping and efficient molecular breeding [11, 37]. However, genetic mapping of *C. maxima* is still in its infancy, with only three low-resolution genetic maps reported [38–40]. Recent advancements in high-throughput genotyping technologies, such as genotyping-by-sequencing (GBS) [41], have provided rapid, efficient and cost-effective genotyping approaches, which have proven their efficiency in saturated genetic map construction and gene/QTL mapping in a variety of different species [42, 43].

Dwarf vine is an important agronomic trait targeted for selection in pumpkin breeding due to its contribution to yield and labor-saving in management and harvesting. In tropical pumpkin (*C. moschata* Duch.) and squash (*C. pepo* L.)*,* the bush type (dwarf vine) is controlled by a dominant gene, and some bush-type related genes and tightly linked markers have been reported [44–50]. In *C. maxima*, the dwarf vine is possibly mainly controlled by two recessive genes which are orthologous to the dwarf genes in *C. pepo*, and might also be regulated by other minor genes [51, 52]. Recently, a *C. maxima* dwarf mutant was reported and the underlying gene was roughly mapped using AFLP markers to a region with a genetic distance of 11.2 cM [53]. Four dwarf-related transcript derived fragments (TDFs), which were involved in cytokinin or indole acetic acid signaling, were identified [53]. The genes controlling dwarf traits in other Cucurbitaceae crops such as melon and cucumber have also been fine mapped recently [30, 54]. Although many studies have been reported on dwarf traits in Cucurbitaceae, to date no dwarf genes have been cloned.

In this study, a *C. maxima* population with 186 F_2_ individuals was generated from a cross between an inbred line with long vine (vine type), Rimu, and a line with dwarf vine (bush type), SQ026. The vine length in the F_2_ population showed a continuous phenotypic variation. The vine length trait was found to be quantitative in nature and the dwarf-type was gibberellin-responsive. The population was then genotyped using the GBS approach, resulting in a total of 458 recombinant bin markers, which were used to create the first high resolution genetic map in *C. maxima* that comprised 20 linkage groups. The map was further used to assist in anchoring and orienting the assembled *C. maxima* genome scaffolds. Moreover, the vine length was measured for individual plants in the F_2_ population and an association analysis was then performed to identify QTLs for the dwarf vine trait. The high-density genetic map we constructed provided an invaluable new tool for genetic research and molecular breeding in *C. maxima*.

## Results

### Sequencing, genotyping, and genetic map construction

Two 96-plex GBS libraries were constructed for the two parents (two replicates for each) and 186 F_2_ plants of the cross Rimu × SQ026. A total of 418 million cleaned reads were obtained, and the number of reads per sample ranged from 0.45 to 4.50 million, with an average of 2.22 million reads for each individual, which was equivalent to ~0.61-fold coverage of the *C. maxima* genome which was estimated to have a size of approximately 373.9 Mb based on the k-mer analysis of the large-scale Illumina genome sequences. Of these reads, 394.57 million (94.4 %) were mapped to the *C. maxima* genome assembly and used for SNP calling. The resulting SNPs were filtered if there was no homozygous variation found between the two parents. A total of 8,660 SNPs were obtained, among which 1,881 SNPs had less than 20 % missing data and a minimum allele frequency (MAF) ≥ 0.2. These 1,881 segregating SNPs were used for genetic linkage map construction and scaffold anchoring, which yielded a genome-wide SNP density of ~1 SNP/144.3 kb.

**Fig. 1:**
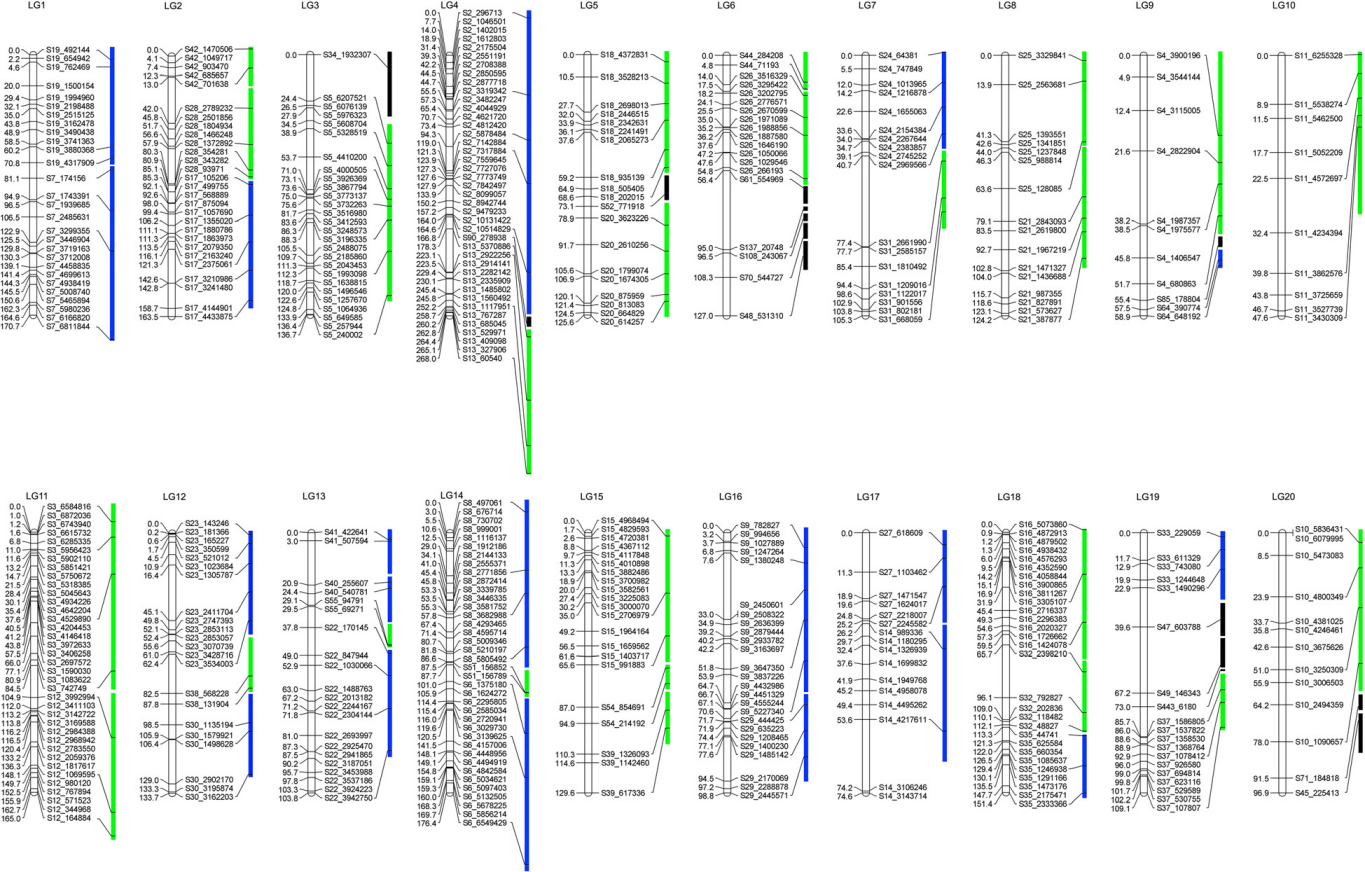
Anchoring of the *C. maxima* assembled scaffolds to the Rimu × SQ026 F_2_ genetic map. Open bars represent the 20 *C. maxima* linkage groups (LGs); SNPs are located on the LGs according to the genetic distance (cM). Pumpkin genome scaffolds (filled bars) are positioned in each LG with corresponding genetic markers. Blue, scaffolds in positive orientation; green, scaffolds in negative orientation (reverse complement); black, scaffolds that were anchored but not oriented

Due to the relatively small size of the mapping population (*n* = 186), many of the 1,881 SNPs co-segregated in the F_2_ population, suggesting that these loci were in close proximity and had not been resolved by recombination events. Therefore, these SNPs were further concatenated into 466 bin markers, with SNPs in one bin considered as a single haplotype. Finally, a total of 458 of the 466 bin-markers were mapped to create the 20 linkage groups (LGs) that corresponded to the 20 chromosomes of pumpkin, with a mean of 22.9 markers per LG. The LGs had an estimated total genetic length of 2,566.8 cM and an average of approximately 5.60 cM per bin (Fig. 1, Additional file 1: Figure S1). The distance between neighboring bin markers ranged from 0.05 cM to 44.89 cM. Based on the estimated size of the pumpkin genome (373.9 Mb), the map defined herein represented an average physical interval of 816.4 Kb per marker, making it the most saturated genetic map of *C. maxima* to date. The size of LGs ranged from 47.6 cM (LG 10) to 268.0 cM (LG 4) with the number of bin markers per LG ranging from 10 to 41. In this map eight large gaps (≥18 cM) between scaffolds in LGs 2, 3, 6, 7, 11, 15, and 19 were detected (Fig. 1).

Segregation distortion plays a dominant part in the plant genome evolution [55]. In our map two segregation distortion regions (SDRs) were detected in LGs 3 and 12, respectively (*P* < 0.05) (Additional file 2: Table S1). The SDR in LG 12 spanned relatively large fraction of the LG, from 0.6 to 16.4 cM (Fig. 1), with marker alleles associated with the Rimu parent. Marker alleles within the SDR of LG 3 skewed toward the SQ026 parent.

### Scaffold anchoring and orienting

The map was further used to anchor and orient the assembled genome scaffolds of the maternal parent of the F_2_ population, Rimu, to the 20 LGs. Altogether, 58 scaffolds with a total length of 194.5 Mb, accounting for 71.7 % of the assembled 271.4 Mb sequences were successfully anchored (Table 1, Fig. 1). The number of scaffolds anchored per LG ranged from 1 (LG10) to 7 (LG6) with sizes ranging from 6.34 Mb (LG10) to 17.91 Mb (LG4). On average, each scaffold contained eight markers, and 44 scaffolds (183.03 Mb; 67.4 % of the assembled scaffolds) were anchored by at least two different markers, which could be oriented with high confidence on the genetic map (Table 1, Fig. 1). For the remaining 14 smaller scaffolds (most of them less than 1.5 Mb and a total of 11.5 Mb) that had only one genetic marker, their orientation on the map could not be determined. Although the average marker distance was 424.7 kb, large gaps were present on the physical map, e.g., a 2.4-Mb gap in scaffold S13 on LG 4 between S13_5370886 and S13_2922256 and a 1.6-Mb gap in scaffold S32 on LG 18 between S32_792827 and S32_2398210.

**Table 1:** Anchoring of *C. maxima* genome assembly to the genetic map

LG	LG size (cM)	No. anchored scaffolds	Anchored scaffold size (Mb)	% of assembly	No. oriented scaffolds	Oriented scaffold size (Mb)	Recombination rate (cM/Mb)
1	170.74	2	11.41	4.20	2	11.41	14.96
2	163.50	3	10.10	3.72	3	10.10	16.19
3	136.74	2	9.42	3.47	1	6.89	19.85
4	268.01	3	17.91	6.60	2	17.52	15.30
5	125.60	3	10.10	3.72	2	9.12	13.77
6	126.97	7	8.03	2.96	2	5.19	24.46
7	105.35	2	6.84	2.52	2	6.84	15.40
8	124.16	2	7.92	2.92	2	7.92	15.68
9	58.93	3	8.25	3.04	2	7.83	7.53
10	47.61	1	6.34	2.34	1	6.34	7.51
11	164.98	2	12.97	4.78	2	12.97	12.72
12	133.66	3	9.42	3.47	3	9.42	14.19
13	103.82	4	8.50	3.13	4	8.50	12.21
14	176.36	3	14.49	5.34	3	14.49	12.17
15	129.60	3	8.17	3.01	3	8.17	15.86
16	98.78	2	9.82	3.62	2	9.82	10.06
17	74.59	2	8.98	3.31	2	8.98	8.31
18	151.41	3	10.26	3.78	3	10.26	14.76
19	109.07	5	7.36	2.71	2	4.92	22.17
20	96.94	3	8.24	3.04	1	6.34	15.29
Total	2566.82	58	194.53	71.67	44	183.03	13.19

### QTL identification for dwarf vine

**Fig. 2:**
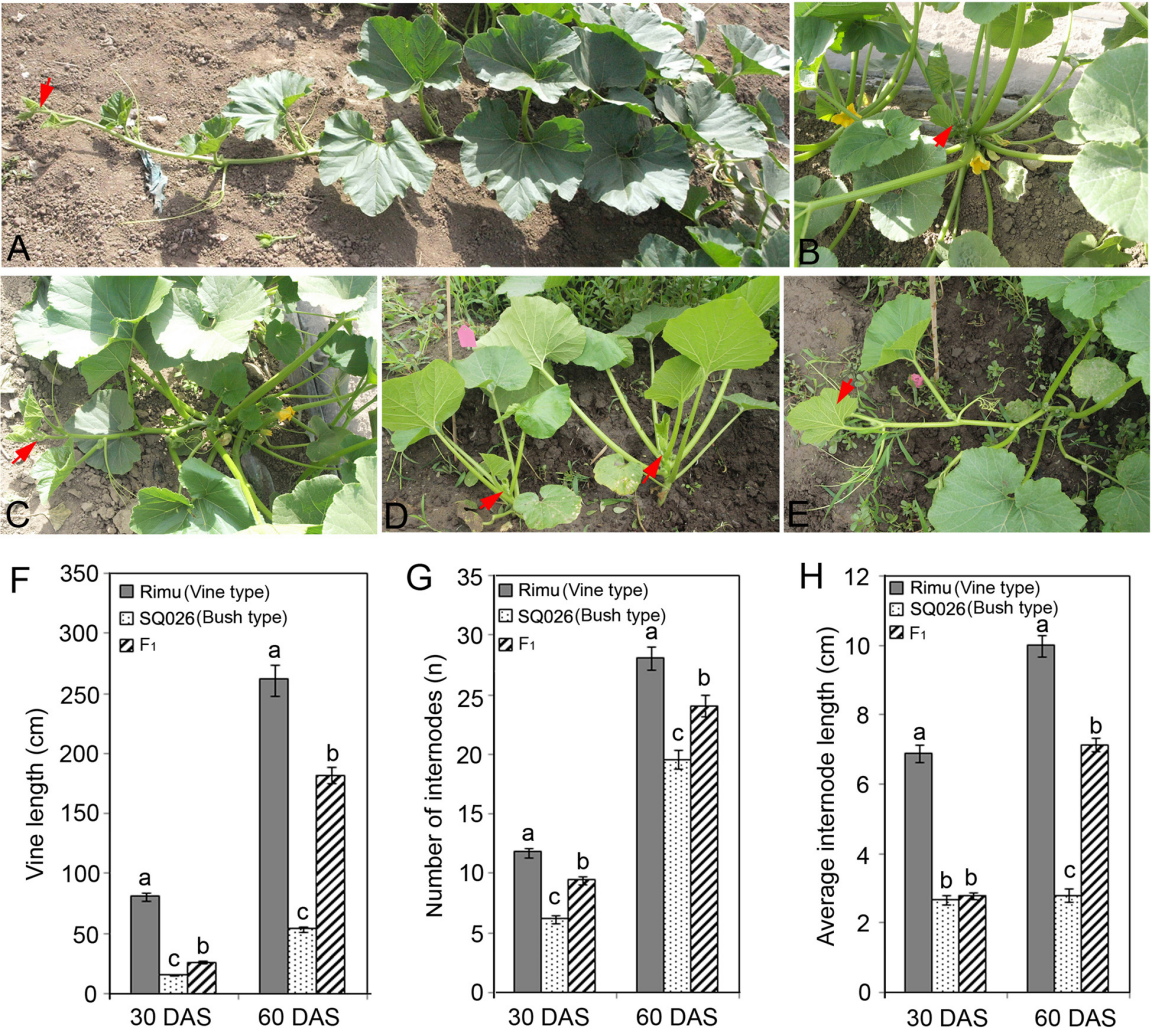
Vine length of the two parents and the F_1_ and F_2:3_ lines. **a**, the inbred line Rimu; **b**, the inbred line SQ026; **c**, F_1_; **d**, the representative F_2:3_ bush type line; **e**, the representative F_2:3_ vine type line. Red arrows point to the vine tips; **f-h**, Vine length, internode number and the average internode length at 30 and 60 days after sowing (DAS). Bars show mean ± SE (*n* = 20). Different letters indicate significant difference at *P* < 0.05

**Fig. 3:**
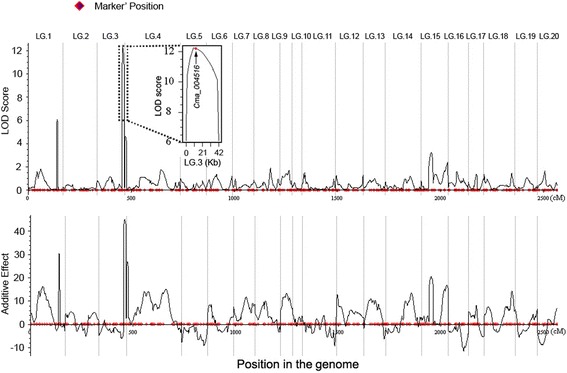
Mapping of QTLs controlling vine length in the F_2_ population and the location of *qCmB2*. Curves in plot indicate the genetic coordinate and LOD score (top) or additive effect (bottom) of detected QTLs. The box inside shows the zoom-in view of the peak on LG 3. Red dot indicates the physical position of the gene *Cma_004516*

The maternal line Rimu and the male parent SQ026 had mean vine length of 256 cm and 73 cm at the 25th internode, respectively, and the F_1_ plants presented an intermediate vine length of 154 cm (Fig. 2, Additional file 2: Table S2). The dwarf SQ026 plants produced shorter vines, as well as fewer and shorter internodes than the vine type Rimu and F_1_ plants at 30th and 60th day after sowing (DAS) (Fig. 2f-h). Transgressive segregation was observed in the F_2_ population for vine length and the average value skewed toward the long vine parent. Frequency distribution of vine length among the test lines is presented in Additional file 1: Figure S2. Using joint analysis (LOD ≥ 4), three QTLs associated with dwarf vine were detected on LGs 1 (*qCmB1*), 3 (*qCmB2*), and 4 (*qCmB3*), respectively (Table 2, Fig. 3), with additive effects ranging from 26.67 to 44.40 and R^2^ values from 7.65 to 21.39 %. As expected, the elite parental cultivar (SQ026) contributed the dwarf vine alleles at all loci. *qCmB2*, the QTL with the largest effect on vine length, explained 21.39 % of the phenotypic variation and mapped to a region between S5_1064936 and S5_649586, which spanned a genetic distance of about 9.1 cM and corresponded to a physical distance of about 0.42 Mb (Fig. 3). The other two minor QTLs explained 7.65 and 9.95 % of the phenotypic variation, respectively. In addition, when the F_2_ individuals were arbitrarily divided into three groups with short, median and long vines, these three QTLs could still be identified based on a permutation determined LOD threshold of 4.

**Table 2:** QTL analysis for dwarf vine in *C. maxima*

Trait	QTL	LG.	Position (cM)	Left Marker	Right Marker	Physical length (Mb)	LOD score	PVE (R^2^ %)	Add	Dom
VL	*qCmB1*	1	142.0	S7_4699613	S7_4938419	0.24	6.07	9.95	30.33	−4.41
	*qCmB2*	3	125.0	S5_1064936	S5_649585	0.42	12.24	21.39	44.40	−1.80
	*qCmB3*	4	2.0	S2_296713	S2_1046501	0.75	4.60	7.65	26.67	−3.81

*VL* vine length, *PVE* percentage of the phenotypic variation explained by the QTL; Add, positive values of the additive effect indicated that alleles from SQ026 were in the direction of increasing trait score; *Dom* dominant effects

### A candidate gibberellin biosynthesis gene in the major QTL region controlling vine length

**Fig. 4:**
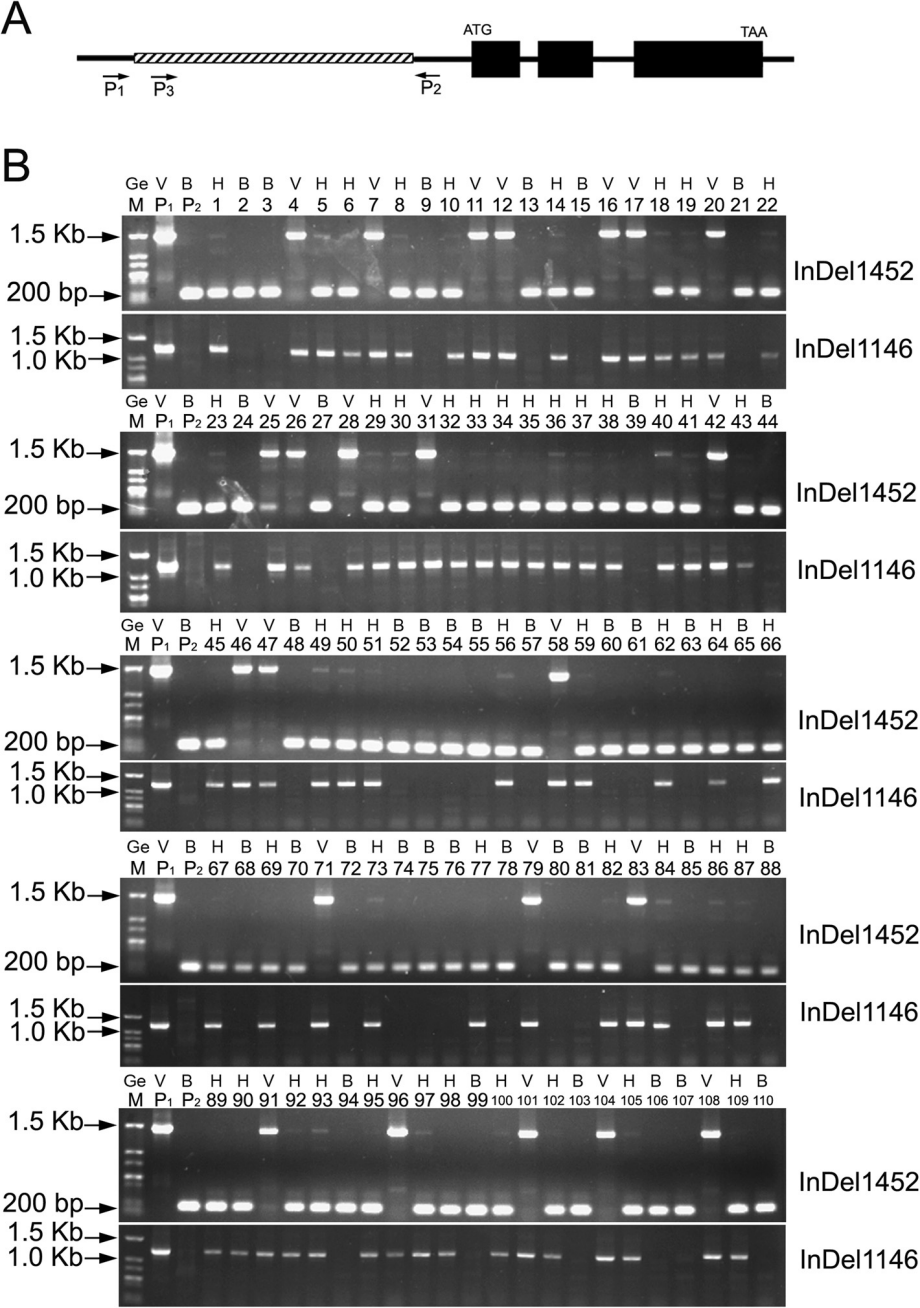
Structure of the gene *Cma_004516* and genotyping of the F_2_ population. **a**, Schematic diagram of gene structure of *Cma_004516.* Solid boxes represent coding regions; open boxes with diagonal lines denotes the deleted fragment of the promoter region of *Cma_004516* in bush type lines; bold lines, introns or 5′, 3′- UTRs. Locations of the indel-markers InDel1452, amplified using the primers P_1_/P_2_, and InDel1146, amplified using the primers P_3_/P_2_, are shown. **b**, Genotyping of the F_2_ population by the two InDel markers. M, DNA marker; P_1_, maternal parent Rimu; P_2_, paternal parent SQ026; 1–110, individual F_2_ lines; Ge, genotypes; V, vine type; B, bush type

The small physical interval of *qCmB2* (~0.42 Mb in length defined by bin-makers S5_1064936 and S5_649585) encompassed 104 predicted protein-coding genes (Additional file 2: Table S3). Many studies have shown that most dwarf or semi-dwarf type varieties were caused by the deficiency of genes involved in the gibberellin (GA) biosynthesis or signaling [56–59]. The dwarf lines in *C. maxima* were GA-sensitive as their vine length was increased significantly after treated with GA (Additional file 1: Figure S3). Among the genes in the genomic region of *qCmB2*, three genes (*Cma_004514*, *Cma_004515* and *Cma_004516*) encoding 2-oxoglutarate (2OG) and Fe(II)-dependent dioxygenase superfamily proteins, which are possibly involved in the GA biosynthesis [60, 61], were identified. We then performed genome resequencing for the male parent, SQ026, and obtained a total of 321.6 million paired-end reads with length of 100 bp. By aligning these reads to the assembled genome of Rimu, the maternal parent, we identified a 1,249-bp insertion/deletion (InDel) (Additional file 1: Figure S4) and two SNPs in the promoter region and a 3-bp InDel and 8 SNPs in the intronic region of *Cma_004516* (Additional file 1: Figure S5). These sequence variations were further verified by PCR cloning and sequencing. Two PCR-based molecular markers targeting this 1,249-bp InDel, named InDel1456 and InDel1146, were developed (Fig. 4a). All F_2_ individuals were genotyped using these two markers (Fig. 4b), and the 1,249-bp InDel polymorphism was found to completely co-segregate with the dwarf vine in the F_2_ and the F_2:3_ populations without any recombinants (Additional file 2: Table S4 and S5). Validation of this InDel polymorphism in 164 pumpkin varieties which are either bush or vine habits showed that the marker could accurately identify the phenotypes (Additional file 2: Table S6). The physical position of the *Cma_004516* gene in the pumpkin genome is 764,230-765,689 bp on the Scaffold S5, which is very close to the peak of LOD curve for the vine length QTL *qCmB2* (Fig. 3).

**Fig. 5:**
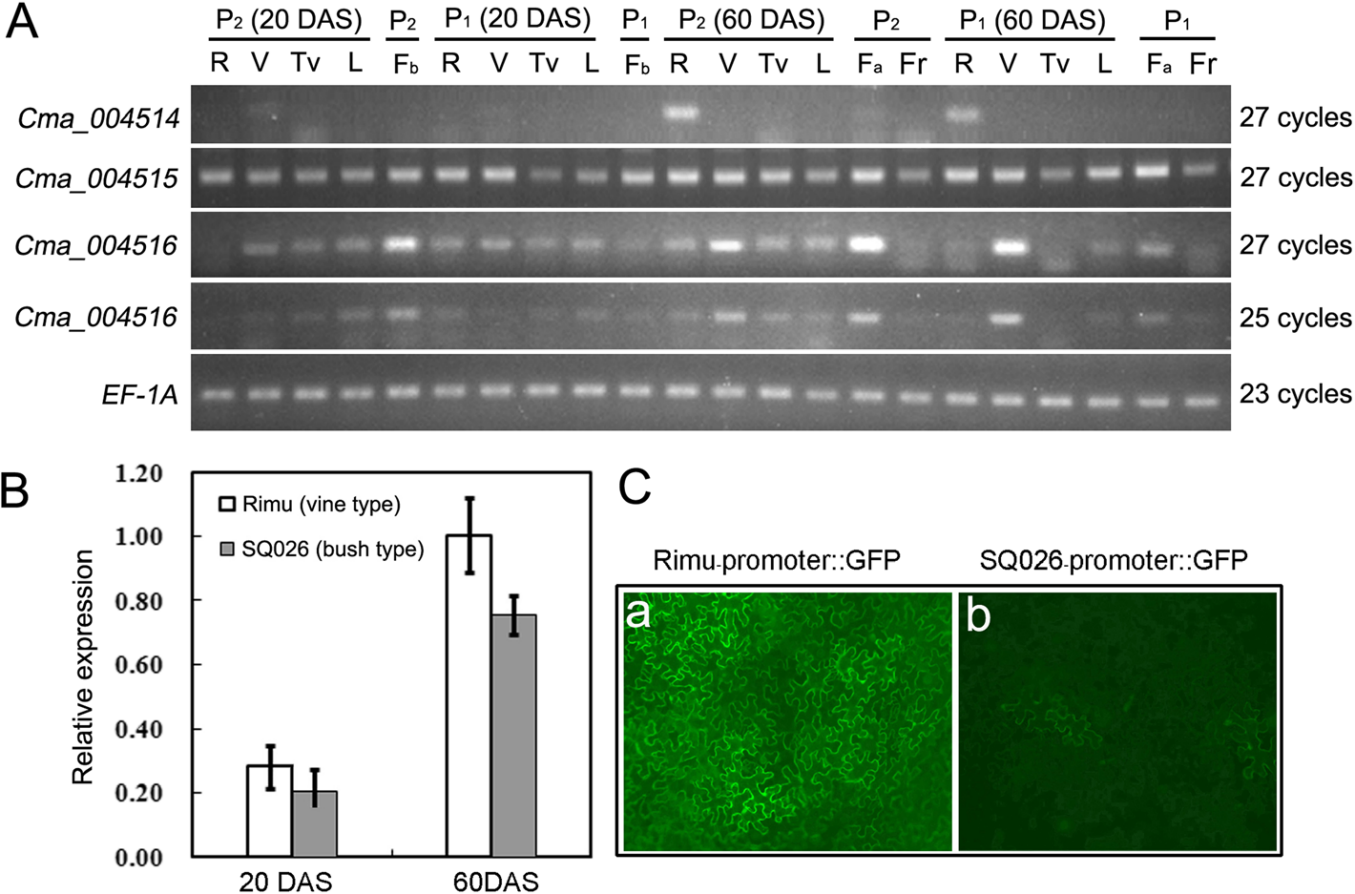
Expression profiles of *Cma_004516* in *C. maxima*. **a**, RT-PCR analysis of *Cma_004516, Cma_004514* and *Cma_004515* mRNA in various tissues. R, roots; V, vines; Tv, tip of the vines; L, leaves; F_b_, flowers before anthesis; Fa, flowers after anthesis; Fr, young fruits; DAS, days after sowing. P_1_, maternal parent Rimu; P_2_, paternal parent SQ026; The right side shows the PCR amplification cycles. *EF-1A* was used as the internal control. **b**, Analysis of *Cma_004516* gene expression in vines by real-time PCR. Each bar represents an average of three independent reactions, including both biological and technical replicates. Error bars indicated SD. **c**, Analysis of the activities of the *Cma_004516* promoters by transient expression in tobacco leaves. a, promoter of the vine type; b, promoter of the bush type

We further investigated the expression pattern of *Cma_004516* with semi-quantitative PCR and qPCR in two parental lines to check whether the expression level was affected by the sequence deletion in the promoter region and contributed to the variance of vine length (Fig. 5a and b). The *Cma_004516* transcripts were highly abundant in elongated vines (60 days after sowing) and flowers (in SQ026), also present in leaves and tips of the vines, and almost undetectable in roots and fruits. The expression level of *Cma_004516* was significantly increased during the vine elongating process (from 20 to 60 days after sowing), and was significantly higher in the vine type lines than in the bush type lines (*p* < 0.05) (Fig. 5a and b). In transient expression assays, the activity of the promoter of *Cma_004516* in bush type line was much weaker than that in the vine type line (Fig. 5c). The expression of another GA 20-oxidase gene in the region of *qCmB2*, *Cma_004514*, was restricted to roots, and another gene, *Cma_004515*, was expressed at the similar level in all tested tissues.

Phylogenetic analysis showed that *Cma_004516* was the ortholog of *MtGA20ox1-B*, the gibberellin 20 oxidase 1-B gene in *Medicago truncatula* (Additional file 1: Figure S6), suggesting Cma_004516 is a possible enzyme that catalyzes the last three steps of the synthesis of active GAs in *C. maxima*. All data present here provides evidences supporting that *Cma_004516* could be a candidate gene controlling vine length in *C. maxima*.

## Discussion

In this study, we described the construction of the first high-density genetic map in *C. maxima* using the bin-markers developed using the GBS technology. This linkage map was further used as a reference to successfully anchor and orient the full genome assembly and to map QTLs for dwarf vine in this species. This map will be valuable for future gene cloning, QTL mapping and marker assisted breeding, and provide a basis for comparative analysis among cucurbit genomes. In addition, the identified QTLs for dwarfism also provide a solid foundation for the characterization of the dwarf gene and uncovering the molecular mechanisms of the dwarfism in pumpkin.

### High-density genetic map construction and genome assembly anchoring in *C. maxima*

The genetic linkage map reported herein is the first map developed using bin markers derived from SNPs in *C. maxima*. Compared to previously reported *Cucurbita* maps [13, 38–40, 62–65], this map had much higher marker density and fewer gaps, making it the most saturated genetic map in *Cucurbita* species reported to date. Furthermore, all bin markers used here possess their unique physical locations in the *C. maxima* reference genome, and are potentially highly transferable among species and even genera, which will facilitate the development of an integrated *Cucurbita* map by merging different maps [31, 64]. The map will also allow comparative genetic studies within this genus and may further elucidate the evolution of different species in the genus. However, several large gaps still exist in this genetic map; therefore, additional markers need to be developed in order to get a better covered map.

Distorted segregation was observed in 21 bin markers, which was lower than that reported in maps constructed in *C. pepo* and from interspecific crosses [13, 62, 63, 65]. Biological segregation distortion can affect a cluster of loci, and form a SDR, which should contain at least three adjacent loci [66, 67]. Based on this criterion, two SDRs were found, one on LG 3 and the other on LG 12 (Additional file 2: Table S1), and both were located near the end of LGs, possibly due to gametic, zygotic or other selections. Other distortion loci scattered along the LGs 2, 4, 5, 7, 11, 14, 15, 16 and 17 were likely a result of non-biological factors, including the bias introduced in the GBS library construction (e.g., highly repetitive regions are much harder to be cloned and sequenced) and missing data points in SNP calling. Marker inconsistency was also observed in a few regions, mostly caused by duplicated marker loci or segregation distortions [68].

Using this map we successfully anchored 58 scaffolds (71.7 % of the 271.4 Mb assembled genome) and oriented 183.0 Mb (67.4 %) of the assembled sequences. Compared to the percentages of anchored and oriented genome sequences in other crop species such as watermelon (93.5 % anchored, 65 % oriented) [11], melon (98.2 %, 90 %) [37], cucumber (72.8 % anchored) [26], cacao (67 %, 50 %) [69], apple (88 %, 66 %) [70], grape (69 %, 61 %) [71], soybean (97 % anchored) [72] and strawberry (94 % anchored) [73], the percentages of anchored and oriented scaffolds in this study were not high. The major factor that limits successful anchoring and orienting of scaffolds is the map density, which depends on the SNP density and the size of the mapping population. Low sequencing coverage and probably the non-optimal restriction enzyme used in the GBS experiment would result in sparse SNP markers. Therefore, additional markers and a larger mapping population would help to generate a higher density genetic map, which can be used to further facilitate anchoring and ordering of the remaining sequences.

### QTL analysis for the bush type vine

Using the map, a major QTL *qCmB2* for dwarfism in *C. maxima* was identified and delimited to a 420-kb physical interval. The QTL contributed to 21.39 % of the phenotypic variation, suggesting the high efficiency in QTL detection using this approach (Table 2, Fig. 3). Among the 104 predicted genes within the QTL region, *Cma_004516* encoded a putative gibberellin 20-oxidase and shared 78.5 and 79.1 % amino acid sequence identity with Arabidopsis gibberellin 20-oxidase 2 and *Medicago truncatula* gibberellin 20-oxidase 1-B, respectively. The physical position of *Cma_004516* was very close to the peak of LOD curve for vine length QTL *qCmB2* (Fig. 3). The SQ026 (bushy parents) allele of *Cma_004516* perfectly co-segregated with the dwarf phenotype across the F_2_ and F_2:3_ populations and a broad range of *C. maxima* germplasm. The large sequence deletion identified in the promoter region of *Cma_004516* possibly caused the decreased expression of *Cma_004516*, which could further decrease the gibberellin level and result in the dwarf phenotype [74, 75]. Therefore, it is reasonable to postulate that *Cma_004516* is the candidate gene for dwarfism in *C. maxima*. However, further experiments are necessary to confirm this hypothesis. In addition, the other two minor QTLs (accounting for about 9.95 and 7.65 % of the phenotypic variance, respectively) also need to be further characterized.

### Dwarf gene in *C. maxima*

Dwarf and semi-dwarf characteristics are important agronomic traits in crop breeding for higher yield. In Cucurbitaceae, dwarf mutants in cucumber [54], melon [30], squash [44], tropical pumpkin [48, 50] and *C. maxima* [53] have been reported, but little is known about the underlying genetic basis of dwarfism. Incomplete dominance or developmental reversal of dominance was observed in the present study. F_1_ plants of the cross between bush and vine plants resembled the bush parent at the early developmental stages while become more like the vine parent at the late stages (Fig. 2). This is consistent with previous observations in *C. pepo* and *C. maxima,* the two species possibly possessing the same dwarf gene [52, 76], while it is contradictory to other previously published reports [44, 48, 50, 53], which indicate that the phenotype of bush habit is a completely dominant trait. Singh [51] reported the length of vine was controlled by two pairs of common dominant genes in *C. maxima*. These inconsistent results are probably due to the fact that bush habits are controlled by multiple different genes or due to the different genetic background of the plant materials used for the studies.

Alternatively, these differences in *C. maxima* could be caused by developmental dominance reversal of the dwarf gene. We provide support for *Cma_004516*, which encodes a GA 20-oxidase, as a candidate gene for dwarfism in *C. maxima*. Gibberellin (GA) deficiency always leads to dwarf or semi-dwarf phenotype [56–59]. The defective GA 20-oxidase genes in rice and Arabidopsis result in the semi-dwarf phenotypes, suggesting that other gene members could supplement GA 20-oxidase activity and contribute partially to the active GA in stem elongation [57, 74]. Therefore, we propose that the developmental reversal of dominance of dwarf gene is caused by the partial overlapping redundancy of GA20-oxidase genes in *C. maxima. Cma_004516* is probably critical for active GA at the early developmental stages, but its function could be compensated by other gene members at the late stages (after 5–10 internodes), resulting in behaving dominantly during the early vine development while recessively during the late vine development. Further evaluation of the GA contents in the bushy plants at different development stages and functional characterization of *Cma_004516* and its closely related genes should facilitate uncovering the regulatory mechanisms underlying this trait.

## Conclusions

Here, we report the construction of the first high-density linkage map in *C. maxima* with bin markers developed using the GBS method, which represents the initial reference map of this species. This map successfully assisted in anchoring and orienting the assembled genome scaffolds, and was further used to detect the quantitative loci controlling vine length in *C. maxima*. A highly possible candidate gene for the major QTL *qCmB2* was identified. This study provides deeper understanding of the molecular mechanisms underlying vine variation in *C. maxima* and will be helpful in accelerating crop improvement in a cost-effective manner through selecting the useful alleles.

## Methods

### Plant materials and phenotyping

Two pumpkin inbred lines, Rimu and SQ026, were used as the parents to generate the F_1_ and F_2_ populations. Rimu is the female parent of ‘Shintosa’, a popular interspecific hybrid rootstock for watermelon and cucumber used worldwide since the 1950s. The SQ026 is bush type with dwarf vine. The 186 F_2_ individuals, 30 F_1_ and 30 parent plants were grown and evaluated in a field nursery at the research farm of Beijing Vegetable Research Centre at Sanya (18.16°N, 109.23°E) in winter of 2014. The vine length and internode length of parents, F_1_, and F_2_ were measured at the adult stage (25th node). In total, 185 F_2_ individuals were evaluated for stem length, due to the loss of an individual resulting from disease.

The F_2:3_ families (progeny of the 185 F_2_ individuals, 30 plants for each family), 201 F_2_ individuals, Rimu, SQ206, F_1_ and 164 selected pumpkin varieties were grown and evaluated in an experimental field under natural environmental conditions at Yanqing (40.4°N, 115.9°E) in the summer of 2015. The vines were characterized at the 10th internode.

### Genotyping by sequencing

Genomic DNA from the 186 individuals in the F_2_ population and the two parents was extracted using a Qiagen plant DNAeasy kit (Qiagen, Valencia, CA) with 100 mg fresh leaf tissue after frozen in liquid nitrogen and grounded into fine powders. The methylation-sensitive restriction enzyme *ApeK*I was used to digest DNA samples. Two 96-plex genotyping-by-sequencing (GBS) libraries were prepared using the protocol described in Elshire et al*.* [41]. The GBS libraries were sequenced on an Illumina HiSeq 2500 system. The TASSEL-GBS pipeline [77] was used to process the GBS sequencing reads for SNP calling. Briefly, raw reads from all samples were combined and collapsed into a master tag list. Only tags occurring at least 10 times were retained and then aligned to the *C. maxima* genomes using BWA [78] with default parameters. Only alignments with mapping quality > = 2 were used for SNP calling.

### Bin-map construction and scaffold anchoring

A bin marker comprised SNPs with the consensus segregation pattern, which did not recombine and were thus incorporated in the bin as described in Ren et al. [10]. The bin-markers were then used for map construction using the JoinMap program v4.0 [79]. Linkage groups (LGs) were identified with likelihood odd (LOD) ratios ≥ 5.0, and marker locations on LGs were graphically displayed using Microsoft Excel via a conditional cell formatting formula and points of disagreement designated as “singletons” were resolved by reassessment of band morphotypes. Scaffolds were then assigned to linkage groups accordingly. When more than one marker had hits on the same scaffold, the scaffold were then oriented on the map.

### Detection of dwarf vine QTLs

Dwarf vine QTLs were identified using the QTL IciMapping v3.1 software, based on the inclusive composite interval mapping (ICIM) model [80]. Threshold values were calculated using 1,000 permutations and QTLs were considered real when ICIM showed the presence of a significant peak at a level of *p* < 0.05. The positions of QTLs were derived based on the peaks from the ICIM scans. The percentage of phenotypic variation explained by each QTL was calculated with a single factor regression (R^2^). The corresponding additive and dominance effects for each QTL were also estimated.

### Genome resequencing of SQ026 and sequence analysis

Genomic DNA was extracted from SQ026 seedlings using the Qiagen plant DNAeasy kit (Qiagen, Valencia, CA). A total of 5 μg of genomic DNA was used to construct a paired-end library with insert sizes of around 250 bp according to the manufacturer’s instructions (Illumina). The library was sequenced on an Illumina HiSeq 2500 system. The raw Illumina reads were first processed to remove adapters and low quality sequences using Trimmomatic [81] and duplicated reads were collapsed into unique reads. The cleaned unique reads were aligned to the reference Rimu genome using BWA [78] with parameters “-n 0.02 –o 1 –e 2” and the paired-end mapping mode. Only alignments with mapping quality > 16 were kept. Following alignments, SNPs and small indels were identified as described in Guo et al. [28] and large structure variations were identified as described in Zhang et al. [82].

### Seedlings treated with GA_3_

Different concentrations (1, 5, 50, 200, 300 and 600 mg/L) of GA_3_ solutions were sprayed on 7-day-old seedlings, and control seedlings were sprayed with double distilled water. Each treatment contained 15 lines, and was replicated five times. Seedlings were treated three times a day for 15 days, then used for picture taken, and the length of the first internode was measured.

### Phylogenetic tree analysis

Sequence alignments were carried out with the ClustalX program [83]. A neighbor-joining phylogenetic tree was created using the MEGA5.0 program [84] with 1,000 bootstrap replications.

### Expression pattern and promoter activity analysis

Total RNA was extracted from different tissues at two developmental stages (20 and 60 days after sowing), except flowers and fruits, which were sampled at the 20th internode, using the Trizol reagent (Invitrogen, California, USA) according to the manufacturer’s protocol, and treated with RNase-free DNase (TaKaRa Biotechnology Co., Dalian, China) to remove residual genomic DNA. Two microgram of total RNA was used for cDNA synthesis using the PrimeScriptII 1st strand cDNA synthesis kit (TaKaRa, Biotechnology Co., Dalian, China) according to the manufacturer’s instructions. Semi-quantitative and quantitative RT-PCR were carried out using the gene-specific primers (Additional file 2: Table S7), and *EF-1A* cDNA was used as the internal control.

A 1,595-bp fragment in the promoter of *Cma_004516* in Rimu (from the start codon to the 1,595 bp upstream of the start codon) and a 752-bp fragment in SQ206 (from the start codon to the 2,001 bp upstream of the start codon, and the 1249-bp deletion) were amplified using the specific primer pairs PL-F/P-R and PS-F/P-R, respectively (Additional file 2: Table S7). After digestion with *BamH*I and *EcoR*I, the fragments were subcloned into the binary vector pYBA1332 upstream of the EGFP coding sequence, in place of the CaMV 35S promoter. The resulting constructs were then transformed into *Agrobacterium tumefaciens* strain EHA105 for transient expression in tobacco as described in Sparkes et al. [85].

## Availability of supporting data

The genome sequence and the SNP marker data will be released to the public through the Cucurbit Genomics Database (http://www.icugi.org) when this paper gets accepted.

## Additional files

Additional file 1
**Figure S1.** Recombination bin-map of the F_2_ population. Bin-map consists of 458 bin markers inferred from 1,881 high quality SNPs in the F_2_ population. Red, Rimu genotype; Green, SQ026 genotype; yellow, heterozygote. **Figure S2.** Distribution of vine length of Rimu, SQ026, F_1_ and F_2_ individuals. **Figure S3.** GA_3_ stimulates vine elongation in SQ026 (bush type). Pictures show plants treated with A, 1 mg/L GA_3_; B, 5 mg/L GA_3_; C, 50 mg/L GA_3_; D, 200 mg/L GA_3_; E, 300 mg/L GA_3_; F, 600 mg/L GA_3_. CK, plants treated with double distilled water. G, Length of the first internodes of the plants after 15-day treatment. *indicates the values are significantly different at *P* < 0.05. **Figure S4.** View of alignments of SQ026 paired-end reads to the Rimu genome around the gene *Cma_004516*. **Figure S5.** Sequence alignment of *Cma_004516* alleles between bush type and vine type lines. Different sequences are shaded in red, introns are underlined, and the gene translation initiation codon (ATG) and stop codon (TAA) are boxed. **Figure S6.** Phylogenetic tree of plant GA20-oxidase family members. Nodes are labeled with the percentage of bootstrap iterations. At, *Arabidopsis thaliana*; Cma, *Cucurbita maxima*; Cs, *Cucumis sativus*; Mt, *Medicago truncatula*; Nt, *Nicotiana tabacum*; Phpa, *Physcomitrella patens*; Sl, *Solanum lycopersicum*; Ta, *Triticum aestivum*; Vv, *Vitis vinifera*; Zm, *Zea mays*; GenBank accession numbers are shown in parenthesis. (PDF 1751 kb) Additional file 2
**Table S1.** Segregation distortion markers. **Table S2.** Phenotypic variation of vine length of Rimu, SQ026, F_1_ and F_2_ population. **Table S3.** Predicted genes in the genome region of the major QTL *qCmB2* on scaffold S5. **Table S4.** Vine phenotypes and genotypes of gene *Cma_004516* in F_2:3_ families. **Table S5.** Vine phenotypes and genotypes of gene *Cma_004516* in the second F_2_ population. **Table S6.** Varieties and their vine phenotypes and genotypes of gene *Cma_004516*. **Table S7.** Primers used in the present study. (XLSX 43 kb)

## Abbreviations

AFLP: amplified fragment length polymorphism
BWA: burrows-Wheeler Alignment tool
CaMV: cauliflower mosaic virus
cM: centiMorgan
cm: centimeters
DAS: day after sowing
EF-1A: elongation factor 1a gene
EGFP: enhanced green fluorescent protein
GAs: gibberellins
GBS: genotyping-by-sequencing
ICIM: inclusive composite interval mapping
InDel: insertion/deletion
Kb: kilobases
LG: linkage group
LOD: likelihood of odd ratio
MAF: minimum allele frequency
Mb: million bases
PCR: poly-merase chain reaction
qPCR: quantitative poly-merase chain reaction
QTL: quantitative trait locus
SDRs: segregation distortion regions
SNP: single nucleotide polymorphism
TASSEL: trait analysis by association, evolution and linkage
TDFs: transcript derived fragments
