# Supplementary material for: A high-density genetic map for anchoring genome sequences and identifying QTLs associated with dwarf vine in pumpkin (Cucurbita maxima Duch.)
Source: BMC Genomics. 2015 Dec 24;16:1101. doi: 10.1186/s12864-015-2312-8 (PMC4690373; doi:10.1186/s12864-015-2312-8)
Supplement: Additional file 1 — Figure S1. Recombination bin-map of the F2 population. Bin-map consists of 458 bin markers inferred from 1,881 high quality SNPs in the F2 population. Red, Rimu genotype; Green, SQ026 genotype; yellow, heterozygote. Figure S2. Distribution of vine length of Rimu, SQ026, F1 and F2 individuals. Figure S3. GA3 stimulates vine elongation in SQ026 (bush type). Pictures show plants treated with A, 1 mg/L GA3; B, 5 mg/L GA3; C, 50 mg/L GA3; D, 200 mg/L GA3; E, 300 mg/L GA3; F, 600 mg/L GA3. CK, plants treated with double distilled water. G, Length of the first internodes of the plants after 15-day treatment. *indicates the values are significantly different at P < 0.05. Figure S4. View of alignments of SQ026 paired-end reads to the Rimu genome around the gene Cma_004516. Figure S5. Sequence alignment of Cma_004516 alleles between bush type and vine type lines. Different sequences are shaded in red, introns are underlined, and the gene translation initiation codon (ATG) and stop codon (TAA) are boxed. Figure S6. Phylogenetic tree of plant GA20-oxidase family members. Nodes are labeled with the percentage of bootstrap iterations. At, Arabidopsis thaliana; Cma, Cucurbita maxima; Cs, Cucumis sativus; Mt, Medicago truncatula; Nt, Nicotiana tabacum; Phpa, Physcomitrella patens; Sl, Solanum lycopersicum; Ta, Triticum aestivum; Vv, Vitis vinifera; Zm, Zea mays; GenBank accession numbers are shown in parenthesis. (PDF 1751 kb) [file 12864_2015_2312_MOESM1_ESM.pdf]

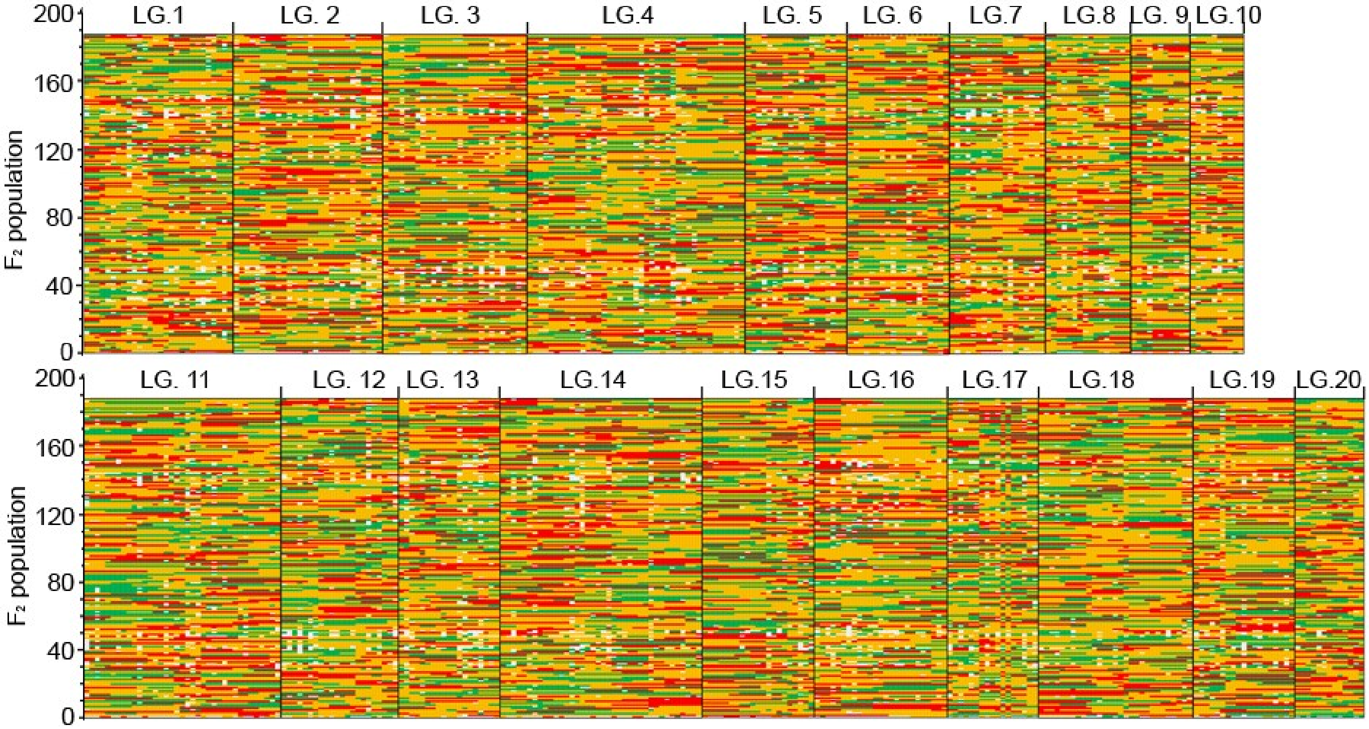

Figure S1. Recombination bin-map of the F_2 population. Bin-map consists of 458 bin markers inferred from 1,881 high quality SNPs in the F_2 population. Red, Rimu genotype; Green, SQ026 genotype; yellow, heterozygote.

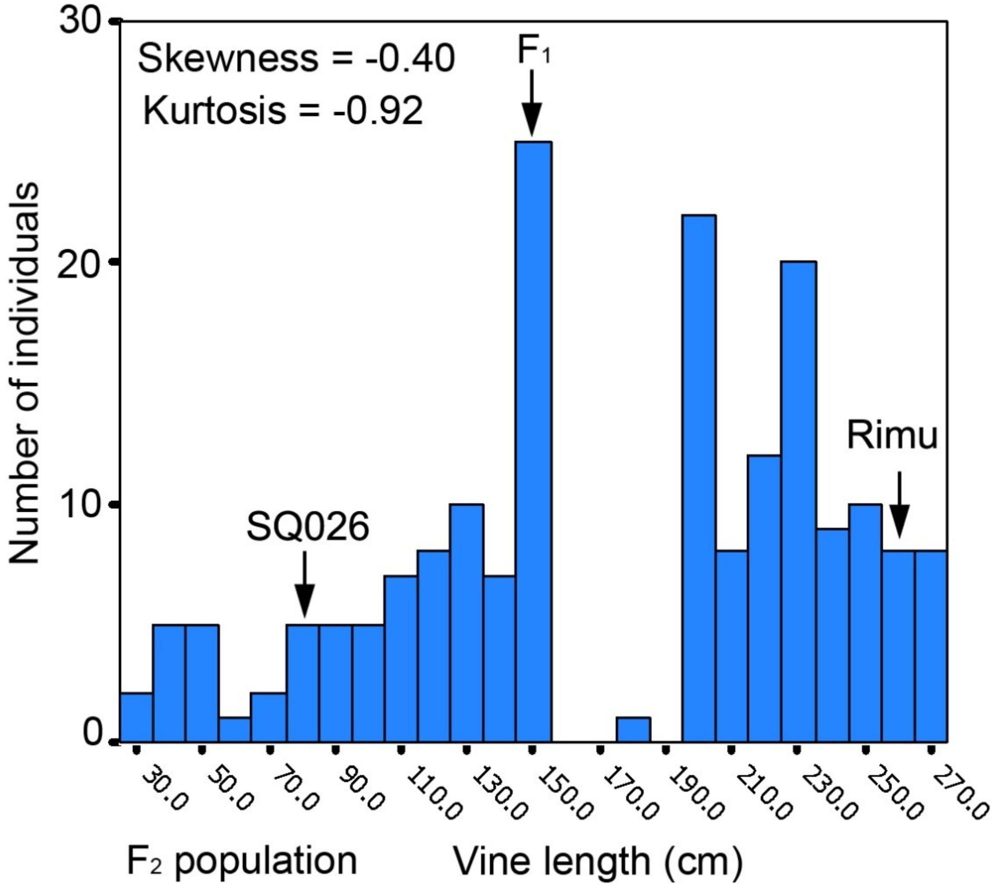

Figure S2. Distribution of vine length of Rimu, SQ026, F_1 and F_2 individuals.

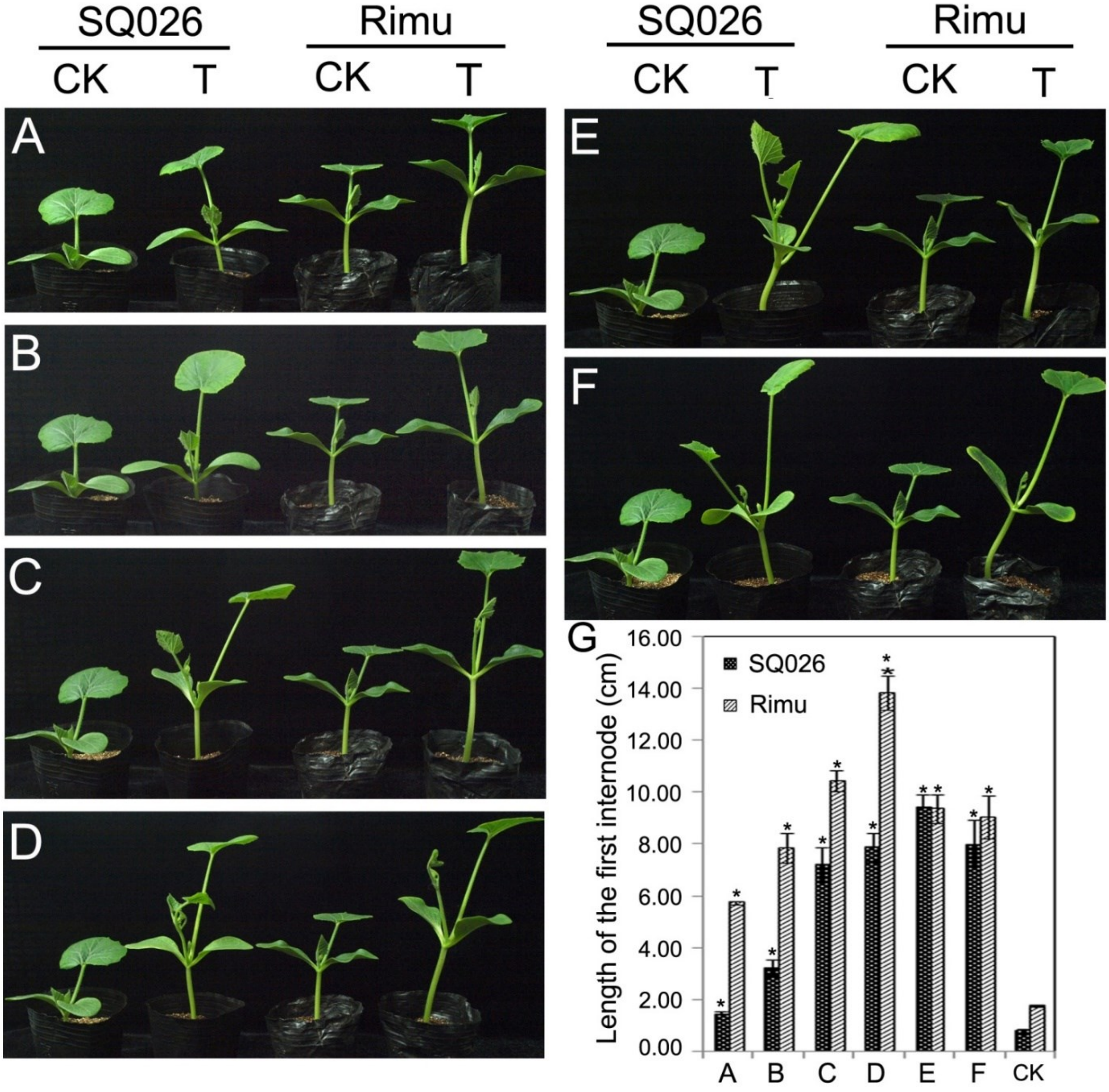

Figure S3. GA_3 stimulates vine elongation in SQ026 (bush type). Pictures show plants treated with A, 1 mg/L GA_3; B, 5 mg/L GA_3; C, 50 mg/L GA_3; D, 200 mg/L GA_3; E, 300 mg/L GA_3; F, 600 mg/L GA_3. CK, plants treated with double distilled water. G, Length of the first internodes of the plants after 15-day treatment. * indicates the values are significantly different at $P < 0.05$.

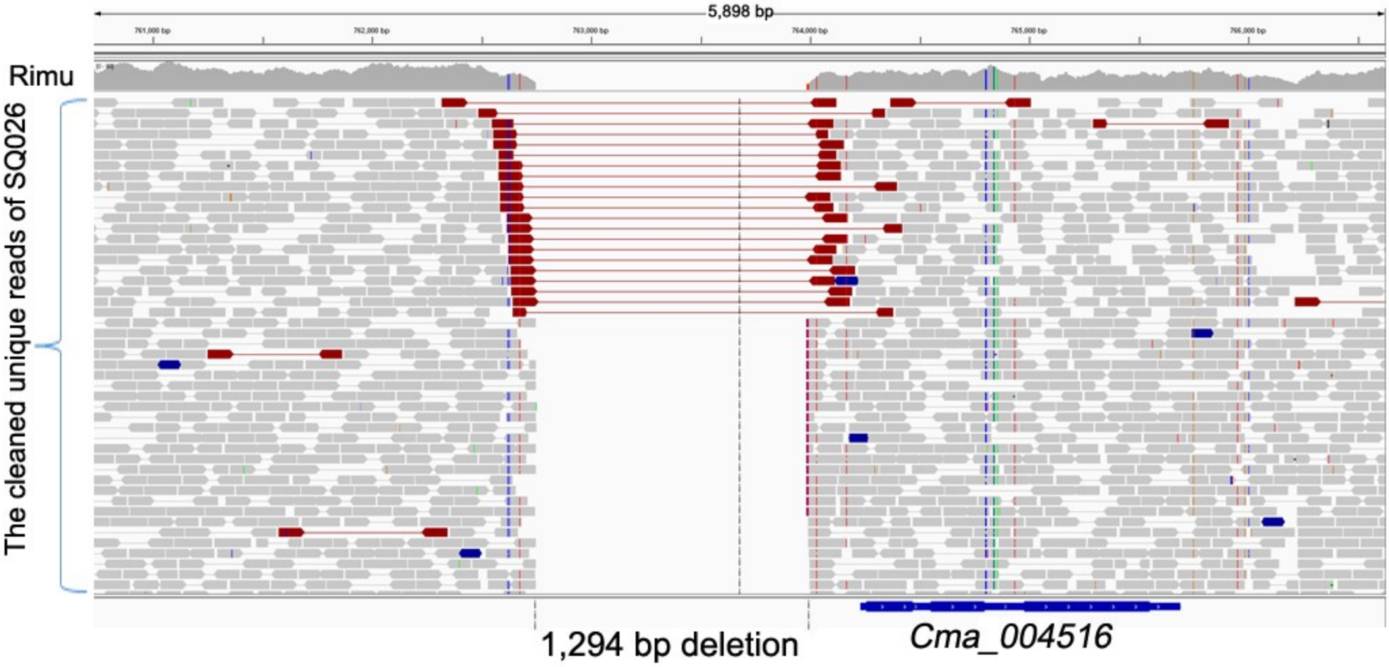

Figure S4. View of alignments of SQ026 paired-end reads to the Rimu genome around the gene *Cma_004516*.

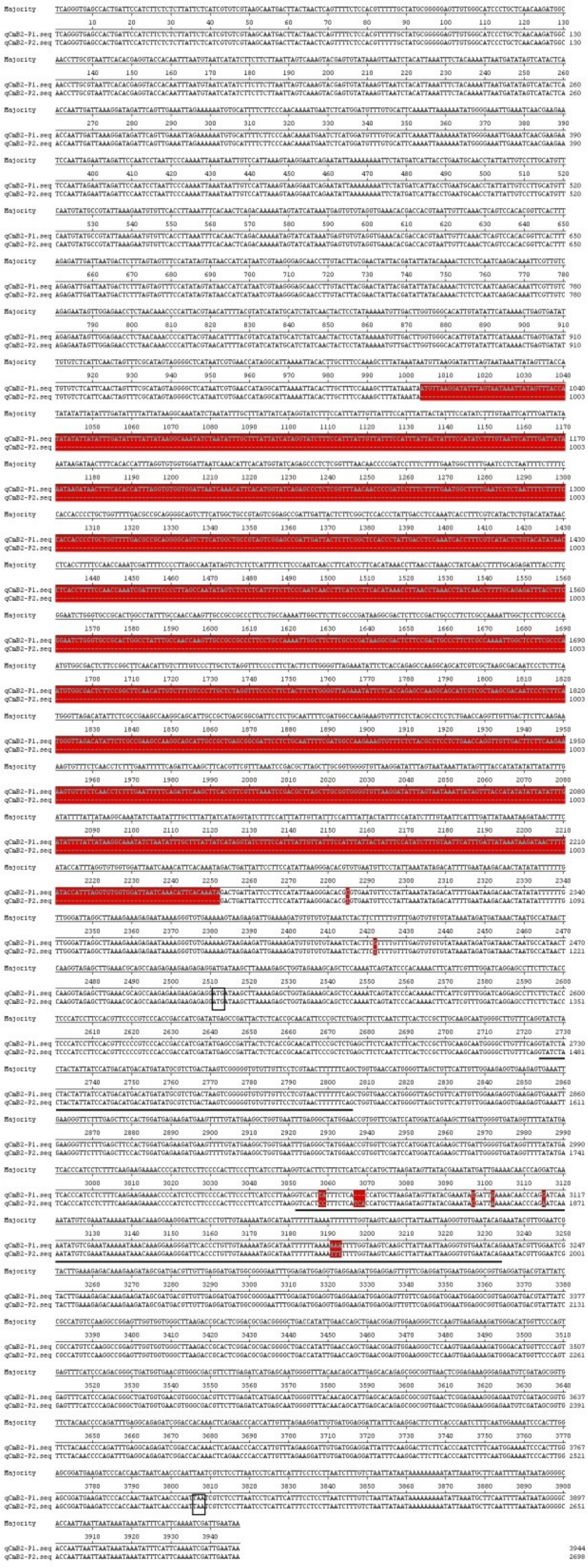

Figure S5. Sequence alignment of *Cma_004516* alleles between bush type and vine type lines. Different sequences are shaded in red, introns are underlined, and the gene translation initiation codon (ATG) and stop codon (TAA) are boxed.

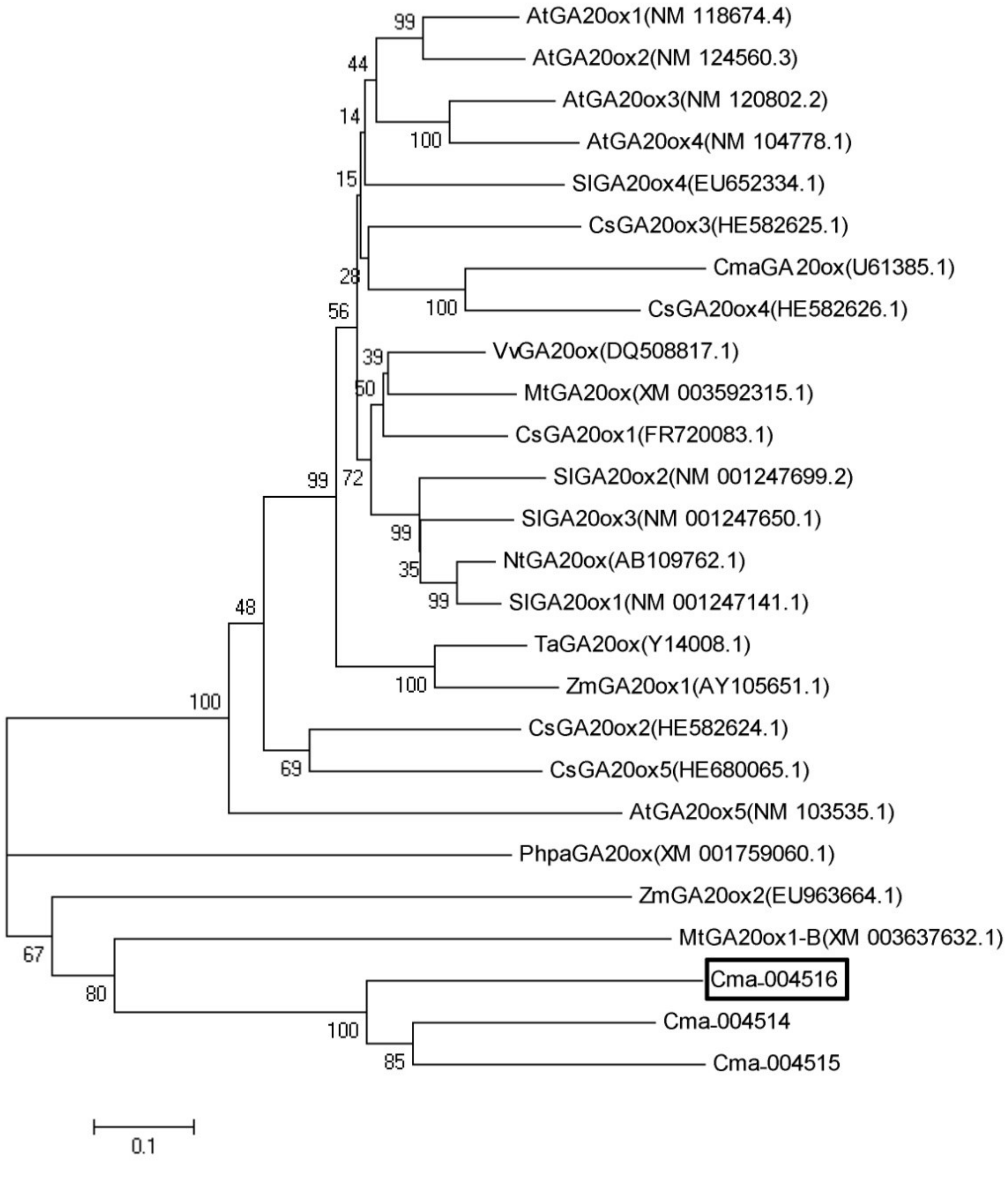

Figure S6. Phylogenetic tree of plant GA20-oxidase family members. Nodes are labeled with the percentage of bootstrap iterations. At, *Arabidopsis thaliana*; Cma, *Cucurbita maxima*; Cs, *Cucumis sativus*; Mt, *Medicago truncatula*; Nt, *Nicotiana tabacum*; Phpa, *Physcomitrella patens*; Sl, *Solanum lycopersicum*; Ta, *Triticum aestivum*; Vv, *Vitis vinifera*; Zm, *Zea mays*; GenBank accession numbers are shown in parenthesis.
